# The levels of triglyceride and total cholesterol in methamphetamine dependence

**DOI:** 10.1097/MD.0000000000006631

**Published:** 2017-04-21

**Authors:** Meijuan Zhang, Dezhao Lv, Wu Zhou, Lili Ji, Beibei Zhou, Han Chen, Yingying Gu, Jiyun Zhao, Jincai He

**Affiliations:** a Department of Clinical Laboratory; b Department of Neurology, The First Affiliated Hospital of Wenzhou Medical University, Wenzhou, Zhejiang, China.

**Keywords:** methamphetamine-dependence, total cholesterol, triglyceride

## Abstract

The serum triglyceride (TG) and total cholesterol (TC) levels have been reported altered in the traditional drug-dependence (such as marijuana and heroin). However, studies assessing the relationships among serum TC, TG, and methamphetamine (MA)-dependence have not been described well. In this study, our aim is to explore the serum TG and TC levels in large sample of MA-dependent patients. A retrospective study was conducted in 938 MA-dependent patients who were recruited between February 2, 2008 and March 11, 2013, with social characteristics and drug-dependence history (duration of MA use, routes of drug administration, and daily dose were collected). Then, the serum levels of TC, TG, glucose (GLU), body mass index (BMI), and blood pressure were measured among the participants. Meanwhile, 985 age- and gender-matched healthy people in the physical examination center were selected as control group. Compared with the control group, significant decreases of TC, TG, GLU, and BMI were observed in MA-dependent patients (*P* < 0.05). Besides, we found that the daily dose of MA use was associated with TC (β = −0.079, *P* = 0.015) and the duration of MA use was independently related to BMI (β = −0.071, *P* = 0.031). This study demonstrated that the levels of TC, TG, GLU, and BMI factors altered in the MA-dependent patients. In addition, there is a negative association between MA dependence and TC and BMI.

## 1 Introduction

Methamphetamine (MA) is also known as methylamfetamine, *N-*methylamfetamine, desoxyephedrine, phenylisopropylmethylamine, and *N*,*a*-dimethylphenethylamine.^[1]^ MA is a new psychostimulant that is widely abused in the world following the heroin, marijuana, and other drugs. MA has been used therapeutically to treat exogenous obesity and attention deficit disorder in clinical settings.^[2]^ However, it is more often associated with a psychostimulant that causes many serious social and health problems.^[3–5]^ Long-term consumption of MA causes economic, social, and health injuries. Research have indicated the toxicology of MA, such as neurotoxicity,^[6,7]^ hepatic damage,^[8]^ cardiac injury,^[9]^ renal insufficiency,^[10,11]^ and cerebral infarcts.^[12]^

Lipids may play some roles in the central nervous system functions that are associated with drug addiction. To date, serum lipid is known to influence relapse of heroin use.^[13]^ Previous research have studied the changes of serum lipid level in heroin and opium abusers. Divsalar et al^[14]^ found that compared to the control group, heroin-dependents showed significantly higher total cholesterol (TC) levels. Kouros et al^[15]^ said that no significant difference of TC level was found in opium- and heroin-dependents, while both heroin- and opium-addict groups showed a significant decrease in serum triglyceride (TG) levels. What is more, the alteration of lipid level with injection of MA had been observed in animal experiment. For example, Koriem and Soliman^[5]^ found that rats revealed a significant increase in serum TG after MA injection. There are important discoveries revealed in previous studies, but few researches assessing the serum lipid levels in a large sample size of MA-dependents have been carried out, the relationship between serum lipid and MA craving is unclear. This study was conducted to investigate the levels of serum TG and total TC in MA-dependents. It also sought to evaluate the relationship between the serum lipid level and drug-dependent history factors.

## 2 Methods

### 2.1 Subjects and setting

A total of 938 studied individuals were selected from Sanyang Detoxification Institute, located in Wenzhou, between February 2, 2008 and March 11, 2013. All individuals had to meet the following inclusion criteria: were only using MA; were between 18 and 60 years of age; had a positive results on urine test for MA at admission; had been in the process of withdrawal for 1 to 7 days; signed informed consent; satisfied the Diagnostic and Statistical Manual of Mental Disorders-IV criteria for MA-dependence; the physical examination items included blood lipids, glucose (GLU), body mass index (BMI), and blood pressure. The subjects were excluded if they were infected with acquired immunodeficiency syndrome (AIDS) or suffered from severe diseases. The patients having dependence on drugs other than MA were also excluded.

The 985 normal physical examination people, who were collected from The First Affiliated Hospital of Wenzhou Medical University, were matched for sex and age. Healthy group had no self-reported family psychiatric history and medication history.

All subjects gave written consent with admission for the use of their clinical data and their blood samples for research purposes, which was approved by the institutional review board and the ethics committee of The First Affiliated Hospital of Wenzhou Medical University. Both the patients and controls are Han Chinese according to their identification card.

### 2.2 Measures

#### 2.2.1 Recorded the sociodemographic characteristics and drug-dependence history

We collected the sociodemographic characteristics in 2 groups, and the drug-dependence history (duration of MA use, routes of drug administration, and daily dose) was conducted by the inpatients at admission.

#### 2.2.2 Assessment of serum TC, TG, BMI, GLU, and blood pressure

The levels of serum TC, TG, and GLU were measured on the 2nd day of admission. The participants’ height and weight were measured at admission. The BMI was counted as weight divided by the square of height (kg/m^2^). The right arm blood pressure of seated participants was obtained under resting condition.

### 2.3 Statistical analysis

The Student *t* test, Mann–Whitney *U* test, or χ^2^ test was appropriately conducted to compare the differences between MA-dependent patients and controls. In adjusted analysis, the effect of age and sex was tested by adding these variables to the analysis model as covariates. Stepwise multiple regression analysis was used to examine the influence of clinical features on TC, TG, GLU, and BMI. All data were analyzed by SPSS 19.0 (SPSS Inc, Chicago, IL). Values less than 0.05 were considered statistically significant.

## 3 Results

### 3.1 Sociodemographics of MA-dependent and healthy controls

The results presented in Table 1 showed that there is no difference in age and sex compared to control group. The range of age at onset in MA-dependent patients is 18 to 56 years. The routes of MA administration were smoking (89.74%), intranasal injection (5.77%), oral (1.28%), intravenous injection (0.54%), and others (2.67%). Of the 938 subjects, 762 (81.24%) were males and 176 (18.76%) were females. The average duration of MA use was 55.44 ± 60.85 months (range 1–228 months). The average daily dose was 0.134 ± 0.210 g (range 0.010–5.000 g).

**Table 1:**
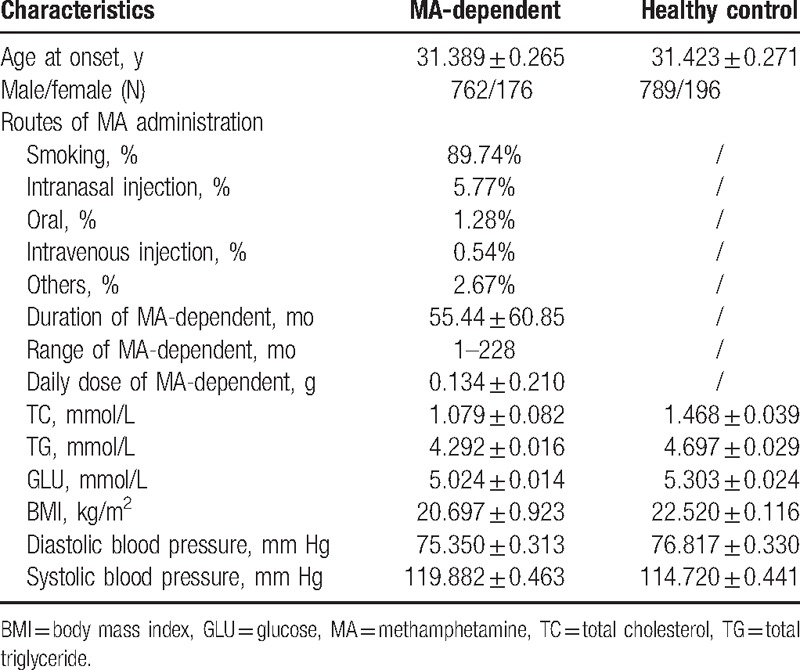
Characteristics between MA-dependents and healthy controls.

### 3.2 TC, TG, GLU, BMI, and blood pressure between MA-dependent patients and healthy control

In adjusted analysis (Table 2), the levels of TC, TG, GLU, and BMI were still significantly increased in MA-dependent patients after age and sex adjustment. However, with the effects of sex and age adding to the analysis of variance as covariates, the significant differences in diastolic blood pressure and systolic blood pressure disappeared (*P* > 0.05).

**Table 2:**
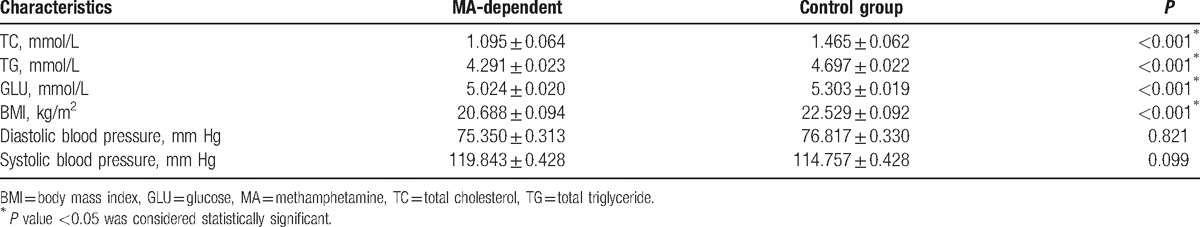
Adjusted levels of TC, TG, GLU, BMI, and blood pressure among the participants.

### 3.3 TC, TG, GLU, BMI, and drug-dependent history among the MA-dependent patients

In the stepwise multiple regression analysis, we took TC, TG, GLU, and BMI as dependent variables separately and sociodemographic variables (gender, age at onset, duration of MA use, routes of drug administration, and daily dose) as independent variables. We found that the daily dose of MA (β = −0.079, *P* = 0.015) had higher risk of lower TC, and the duration of MA use (β = −0.071, *P* = 0.031) was an independent contributor to serum BMI levels. However, gender, age at onset, duration of MA use, routes of drug administration, and daily dose were not found to be associated with serum TG, BMI, and blood pressure (all *P* > 0.05). The results are presented in Table 3.

**Table 3:**
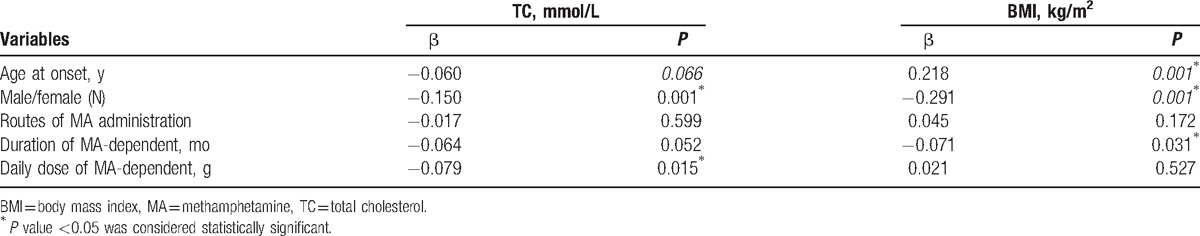
Stepwise multivariate regression analysis when TC and BMI were used as dependent variable, and age at onset, gender, routes of MA administration, duration of MA-dependence, daily dose of MA-dependent patients were used as independent variables.

## 4 Discussion

In this study, we studied the influence of MA-dependence on TC, TG, GLU, BMI, and blood pressure in a large population. We found that the levels of TC, TG, GLU, and BMI were significantly decreased in MA-dependent patients. Besides, we found that the daily dose of MA had a higher risk of lower TC, and the duration of MA use was related to serum BMI level. To the best of our knowledge, this is the first study to investigate the associations between risk factors of serum lipid levels and MA-dependence in a large population.

Compared with healthy controls, we found that MA-dependent patients had lower level of serum TC and TG. While the result of animal experiment performed by Koriem demonstrated that MA-injected rats showed a significant decrease (*P* < 0.05) in TC and TG levels as compared with control rats.^[5]^ Another study carried out by Suriyaprom et al^[16]^ showed no significant difference between MA-dependent and healthy control groups. We thought that the different results may be explained by the shortage of specimens. The levels of GLU and BMI were also significantly decreased in MA-dependent patients, which is in line with our literature published before.^[17]^ Besides, various researches indicated that MA may contribute to lower BMI.^[16,18]^ The decrease in the above indicators could be explained as below: first, MA-dependent patients suffer cognitive deficits and abnormal metabolic activity, which affect nutritional status.^[12,19]^ Second, this condition is further worsened by a drastic reduction in oral health in MA-dependent patients, resulting in improper chewing and poor digestion.^[20]^ The influences of dietary intake on the serum lipid and BMI have been presented in previous literature.^[21]^ Above all, it may be explained why the levels of TC, TG, and BMI decreased in Ma-dependents.

As the results showed in the stepwise multiple regression analysis, the duration of MA use was independently related to BMI and the daily dose of MA use increased a risk of lower serum TC levels. Volkow et al found that significant dopamine (DA) transporter reduced in the striatum and nucleus accumbens of MA users,^[22]^ with DA being one of the neurotransmitters involved in regulating food consumption through modulation of the rewarding properties of food.^[23]^ Considering the fact that the main source of the serum lipid in human body is daily diet and taking the above reasons together, we summed up that the use of MA would reduce the levels of BMI and TC. Besides, consistent with the results of other researchers, we found that BMI to be independently related to age and gender and the TC to be independently related to gender.^[24]^

Compared with healthy subjects, the reduction in lipid levels in MA-users suggests that this population may be in a malnourished state. As we all know, consumption of drugs will increase the risk of infection with AIDS, while malnutrition may impact the course of human immunodeficiency virus (HIV) infection through a variety of mechanisms, including compromising host immune function, diminishing response to therapies, and promoting comorbidities.^[25]^ More studies show that drug users have lower BMI and lower percent fat mass than nondrug users, despite similar or higher dietary intakes.^[18,26,27]^ In addition, at similar levels of BMI, heavier drug use is associated with lower percentage body fat,^[28]^ while weight loss is a significant predictor of mortality.^[29]^ Determining the prevalence of poor nutrition in this population may help us to identify appropriate nutritional interventions to improve quality of life in this vulnerable population and maximize the benefits of antiviral therapy for drug users who are HIV-infected.

The results of characteristics between MA-dependent patients and control group revealed that the routes of MA administration were smoking (89.74%), intranasal administration (5.77%), oral (1.28%), intravenous injection (0.54%), and mixed (2.67%). Compared with the routes of heroin administration,^[30]^ the decrease in the routes of intravenous injection has greatly reduced the spread of kinds of infectious diseases. It is an important reason why MA used as a new-type drug is more and more popular among the drug users. Of the 938 subjects, 762 (81.237%) were males and 176 (18.763%) were females. This result is in line with our previous research.^[31]^ However, some reports indicate that MA was more prevalent among females in Tijuana, Mexico.^[32]^

Finally, the limitations of this paper should be discussed as follows: first, the dates of sociodemographic variables and drug-dependent history were derived from self-report of patients, it may have caused a biased influence on the results. Second, measurement error and observer bias were present in this study as a result of it being a retrospective study. Third, as it is a regression study, the differences of the daily diet and inherited diseases in drug-abuse individuals could not be recorded for details, which leads to deviation from the actual situation. Last but not least, the lack of large-scale surveys in other kinds of traditional drugs (opium, heroin, marijuana, etc.) makes it impossible to elaborate the difference between the new-type drug and the traditional drugs clearly. Further studies should be conducted without the above defects.
